# Differential Apoptotic and Mitogenic Effects of Lectins in Zebrafish

**DOI:** 10.3389/fendo.2019.00356

**Published:** 2019-06-05

**Authors:** Kaidi Wang, Chengdong Liu, Yiying Hou, Huihui Zhou, Xuan Wang, Kangsen Mai, Gen He

**Affiliations:** ^1^Key Laboratory of Mariculture, Ministry of Education, Ocean University of China, Qingdao, China; ^2^Laboratory for Marine Fisheries Science and Food Production Processes, Qingdao National Laboratory for Marine Science and Technology, Qingdao, China

**Keywords:** lectins, proliferation, apoptosis, zebrafish, metabolism

## Abstract

Plant lectins represent a major group of anti-nutritional factors that can be toxic to human and animals. However, the mechanisms by which lectins regulate cell fates are not well-understood. In the present study, the cellular and molecular impacts of three common lectins, agglutinins from wheat germ [wheat germ agglutinin (WGA)], soybean [soybean agglutinin (SBA)], and peanut [peanut agglutinin (PNA)] were examined in zebrafish embryo and liver cells. WGA and SBA were found to induce cell apoptosis both *in vitro* and *in vivo*, while PNA stimulated cell proliferation. WGA and SBA reduced levels of B cell lymphoma-2 (Bcl-2), phosphorylation of Bcl-2-associated death promoter (Bad), cyclin-dependent kinase 4 (Cdk4), and phosphorylation of the retinoblastoma (Rb). WGA and SBA also inhibited the activities of cell survival pathways including protein kinase B (Akt), extracellular signal-regulated protein kinases 1 and 2 (Erk1/2), and target of rapamycin (Tor). Furthermore, WGA and SBA shifted the cellular metabolism characterized by reduced expression of glucose-6-phosphate dehydrogenase (*g6pd*) and increased expression of glutamine synthetase (*glul*) and glutamate dehydrogenase (*glud*). However, PNA showed the opposite effects toward these molecular markers compared to those of WGA and SBA. Therefore, our results revealed some plant lectins (WGA and SBA) were toxic while the other (PNA) was mitogenic. Further characterization of the distinct functions of individual lectins should be valuable for both nutrition and other potential applications.

## Introduction

Lectins are carbohydrate binding proteins found in most plants and can act as a major anti-nutritional factor (ANF) that decrease the bioavailability of nutrients and cause adverse physiological effects in animals (1–3). The presence of significant amounts of lectins in foods causes occasional public poisonings and leads to inferior performances in animal husbandry (4, 5). For example, soybean agglutinin (SBA) is known to be a major contributor of the growth-inhibitory effect of soybean meal in chickens (6), rats (7), and salmonids (8). Full characterization of the physiological effects of lectins is much warranted for public safety and agriculture.

Some lectins are known to be very resistant to common digestive proteases and not degraded during their passage through the digestive tract (9). They can be taken up into the intestinal epithelial cells and transported throughout the body (9, 10). Lectins, such as SBA, concanavalin A (Con A, from *Canavalia ensiformis*), and phytohaemagglutinin (PHA, from *Phaseolus vulgaris*) have been shown to inhibit various digestive enzymes, such as enterokinase, dipeptidase, alkaline phosphatase, etc. in chickens and rats (11, 12). SBA was known to bind to the intestinal mucosa and lead to disruption of brush borders and reduction of nutrient absorption in Atlantic salmon, rainbow trout, and piglets (8, 13). Furthermore, an appreciable portion of lectins, such as WGA (wheat germ agglutinin) and SBA is transported across the gut wall and cause systemic effects through circulation in rats (14, 15). Liver and pancreas hypertrophy were frequently observed in rats fed with diets containing SBA, WGA, and PNA (peanut agglutinin) (16–18). Disrupted hormonal and metabolic homeostasis was proposed as causative reasons in rats (19), but the exact mechanisms are not clear.

Although the pathological effects of plant lectins are well-documented (12, 14, 17), the exact molecular reactions exerted by lectins toward targeted cells and tissues still remain largely unexplored. Rather, the cytotoxic effects of lectins are well-studied in cancerous cells (20–22). For example, Con A has been found to induce apoptosis by increasing cytochrome c release and caspase-9 and caspase-3 levels in human melanoma A375 cells (23). SBA also elicited apoptosis, autophagy, and DNA damage in HeLa cells (24). However, most of these studies were focused on the changes of apoptotic machinery. The upstream signaling that trigger lectin-mediated apoptosis was not well-understood. Furthermore, whether the pro-apoptotic effect is a general character of lectins remains an open question.

To date, the molecular effects of some lectins on aquatic animals were very scarce, regardless of their confirmed toxicity in aquaculture. Growth reduction was observed in rainbow trout fed SBA at concentrations that could be found in commercial diet (25). SBA influenced intestine as well as internal organs, such as pancreas (8, 25–28). Further characterization of lectin-mediated effects and their underlying mechanism is becoming increasingly valuable because of increased inclusion of plant proteins in aquafeeds (29, 30), which make lectins a practical threat to aquatic animals.

The objective of this study was to investigate the cellular effects and molecular mechanism of three lectins (SBA, WGA, and PNA) using zebrafish embryo and liver cells. These lectins were from the most commonly used plant proteins (soybean, wheat, and peanut meals) in aquafeeds.

## Materials and Methods

### Experimental Animals

Wild-type zebrafish (*Danio rerio*) was maintained at 28°C with a 14/10 h light/dark cycle and fed twice daily. Fertilized eggs were obtained by natural breeding. The embryos were staged according to the standard method (31). All experiments were conducted in accordance with the guidelines approved by the Animal Care Committee of Ocean University of China (Permit Number: 11001).

### Effects of Lectins on Embryo Morphogenesis and Viability

Fertilized embryos were collected and placed into 24-well-culture plates at a density of 15 embryos per well. SBA (Sigma, # L1395), WGA (Sigma, # L9640), or PNA (Sigma, # L0881) was added at 4 hpf at the concentrations of 500 μg/ml dissolved in E3 media (5 mM NaCl, 0.33 mM MgSO_4_, 0.33 mM CaCl_2_, 0.17 mM KCl). The embryos were kept at 28.5°C until 48 hpf. The apoptotic cells in zebrafish embryo were visualized by Acridine Orange (AO) staining. Specifically, the embryos were dechorined manually and placed into E3 media containing 5 μg/ml AO (Sigma, # A6014) for 1 hr. After staining, the embryos were washed three times in E3 media before image acquisition. Tricaine were used to immobilize the embryos. Images were captured using a microscope (Nikon Eclipse Ti-S) equipped with a Nikon DS-U3 camera. NIS-Elements software was used.

### Cell Cultures and Treatments

Zebrafish liver (ZFL) cell line was obtained from China Center for Type Culture Collection and maintained in medium containing 50% Leibovitz's L-15 (Sigma, # L1518), 35% Dulbecco's modified Eagle's medium (DMEM; GIBCO, # 12100046), and 15% Ham's F-12 (GIBCO, # 21700075) supplemented with 10% heat-inactivated fetal bovine serum (GIBCO, # 10091148), 0.15 g/l sodium bicarbonate (GIBCO, # 25080094), 15 mM HEPES (GIBCO, # 15630080), 0.01 mg/ml insulin (GIBCO, # 41400045), 50 ng/ml epidermal growth factor (GIBCO, # PHG0311), and 2 mM GlutaMAX (GIBCO, # 35050061) at 28°C.

For proliferation detections, after 2 days of cells culture, cells were starved in serum-free medium for 12 h and changed to experimental medium (1% FBS, 0.15 g/l sodium bicarbonate, 15 mM HEPES, 0.01 mg/ml insulin, 50 ng/ml epidermal growth factor, and 2 mM GlutaMAX) with 1, 2, 5, 8, 10 μg/ml PNA for 48 h before lysis. Cells without lectin treatment were served as the control. All experiments were repeated at least three times.

### MTT Assay

For the 3-(4, 5-dimethylthiazol-2-yl)-2,5-diphenyltetrazolium bromide (MTT) assay, ZFL cell suspension was seeded in 96-well-culture plates containing 100 μl of mix medium at the density of 10^5^ cells/ml and attached to the wells for 24 h. Cells were treated with various concentrations of WGA at 0, 5, 10, 20, 50, 100 μg/ml for 9 h, SBA at 0, 0.3, 0.6, 0.9, 1.2, 1.5 mg/ml for 9 h or PNA at 0, 1, 2, 5, 8, 10 μg/ml for 48 h, separately at 28°C. Then, 11 μl of MTT (5 mg/ml) (Sigma, # M2128) was added to each well and incubated for 4 h. Subsequently, the media was discarded and 150 μL of DMSO (Sigma, # D2650) were added to each well and incubated at 28°C for 10 min to dissolve the formazan crystals. Absorbance readings of MTT-formazan products were performed at 570 nm on a microplate reader (Synergy HT, BioTek). Each treatment was duplicated in eight wells, and the experiment was repeated three times. The results were represented as the percentage of average cell viability of the lectin-treated groups to the control group.

### Cell Apoptosis Assay

Cell apoptosis was measured using an Annexin V-FITC/propidium iodide (PI) apoptosis detection kit (Beyotime, # C1062) following the manufacturer's instructions. ZFL cells were seeded in 6-well-culture plates at a density of 10^6^ cells/ml. After 48 h of cultivation, cells were treated with serial concentrations of WGA at 0, 5, 10, 20, 50, and 100 μg/ml or SBA at 0, 0.3, 0.6, 0.9, 1.2, 1.5 mg/ml, respectively, for 9 h. Cells were then trypsinized and centrifuged at 200 g for 5 min and the supernatant was discarded. The cells were resuspended in binding buffer (Beyotime, # C1062-2) at a concentration of 1 × 10^6^ cells/ml and treated with 5 μl Annexin V-FITC for 10 min at room temperature and stained with 10 μl PI for 10 min in the dark. Approximately 10,000 cells from each sample were analyzed using a BD Accuri™ C6 flow cytometer and the apoptotic cells were calculated. The percentage of cells positive for Annexin V-FITC and/or PI was reported inside the quadrants. The right lower quadrant represents annexin V positive/propidium iodide (PI) negative staining (Q1-LR: AV+/PI-) indicated early apoptosis; the right upper quadrant representing both high annexin V and PI staining (Q1-UR: AV+/PI+) indicated late apoptosis; the left upper quadrant represents low annexin V, and high PI staining (Q1-UL: AV-/PI+) indicated necrosis and the left lower quadrant (Q1-LL: AV-/PI-) indicated viable cells. The apoptotic ratio was represented as the percentages of early plus late apoptosis cells.

### Cell Signaling Analyses

To examine the effects of lectins on intracellular signaling, after the complete medium was removed, cells were treated with WGA at 0, 5, 10, 20, 50, 100 μg/ml, SBA at 0, 0.3, 0.6, 0.9, 1.2, 1.5 mg/ml or PNA 0, 1, 2, 5, 8, 10 μg/ml, respectively, for 4 h in serum-free medium. Cells were then stimulated with complete medium containing the same concentration of lectins for 15 min for cell signaling activations. The presence and activities of intracellular molecules involved in different signaling pathways were examined by western blot using correlated antibodies.

For western blot analyses, cells were rinsed twice with ice-cold PBS and lysed with RIPA lysis buffer [50 mM Tris, 150 mM NaCl, 0.5% NP-40, 0.1% SDS, 1 mM EDTA, pH 7.4, with protease and phosphatase inhibitor cocktail (Roche)]. The cell lysates were centrifuged at 12,000 g for 20 min at 4°C and the supernatant was collected. Protein concentration was measured using a BCA Protein Assay Kit (Beyotime, # P0011) according to the manufacturer's instructions with bovine serum albumin as standard. After normalization, samples (10 μg of protein) were separated by SDS-PAGE gel for 1 h at 150 V and then transferred to a polyvinylidene difluoride membrane (PVDF) (Millipore) for 1 h at 100 V. The membrane was blocked with 5% non-fat milk in 20 mM Tris, 150 mM NaCl, 0.1% Tween 20, pH 7.4 (TBST) for 1 h at room temperature and incubated with indicated primary antibodies overnight at 4°C, followed by secondary antibodies for 1 h at room temperature. Enhanced chemiluminescence (ECL) (Beyotime Biotechnology, #P1008) detection was used according to the manufacturer's directions. The following antibodies were used: antibodies against Bcl-2 (CST, # 4223), phospho-Bad (Ser136) (CST, # 4366), Bad (CST, # 9239), Cdk4 (Santa Cruz, # 23896), phospho-Rb (Ser807/811) (CST, # 8516), Rb (CST, # 9313), phospho-Akt (Thr308) (CST, # 13038), phospho-Akt (Ser473) (CST, # 3787), Akt (CST, # 9272), phospho-Erk1/2 (Thr202/Tyr204) (CST, # 4370), Erk1/2 (CST, # 9107), phospho-S6k (Thr389) (CST, # 9205), S6k (CST, # 9206), and β-Tubulin (CST, # 2146). All these antibodies were developed using antigenic regions completely conserved in zebrafish, and many had been successfully used in zebrafish as reported before (32, 33). All experiments were repeated at least three times. The densities of the protein bands were normalized to that of β-Tubulin, which served as an internal control. All the band intensities were quantified using NIH Image 1.63 software.

### Quantitative Real-Time PCR (qRT-PCR)

For qRT-PCR, cells were lysed using Trizol Reagent (Thermo, # 15596018) according to the manufacturer's instructions. The extracted RNA was quantified using a Nanodrop 2000 spectrophotometer (Thermo). The integrity of the RNA was examined using 1.5% agarose gel. cDNA preparation and qRT-PCR analysis were conducted as previously described (34). All primer sequences of target genes are listed in Supplementary Table 1. The expression level of a particular gene transcript was calculated based on the standard curve and normalized by the elongation factor 1 α *(ef1*α*)* levels, as no expression changes of ef1α were observed in liver among different treatments (data not shown). The gene expression levels were calculated by 2^−ΔΔ*CT*^ method (35). The data were reported as fold increases of the control.

## Statistical Analysis

All statistical evaluations were analyzed by one-way analysis of variance (ANOVA) followed by Tukey's multiple range tests using the software SPSS 19.0. Differences were regarded as significance when *P* < 0.05. Each value is expressed as means ± S.E.M.

## Results

### The Influence of Lectins on the Morphogenesis of Zebrafish Embryos

**Figure 1:**
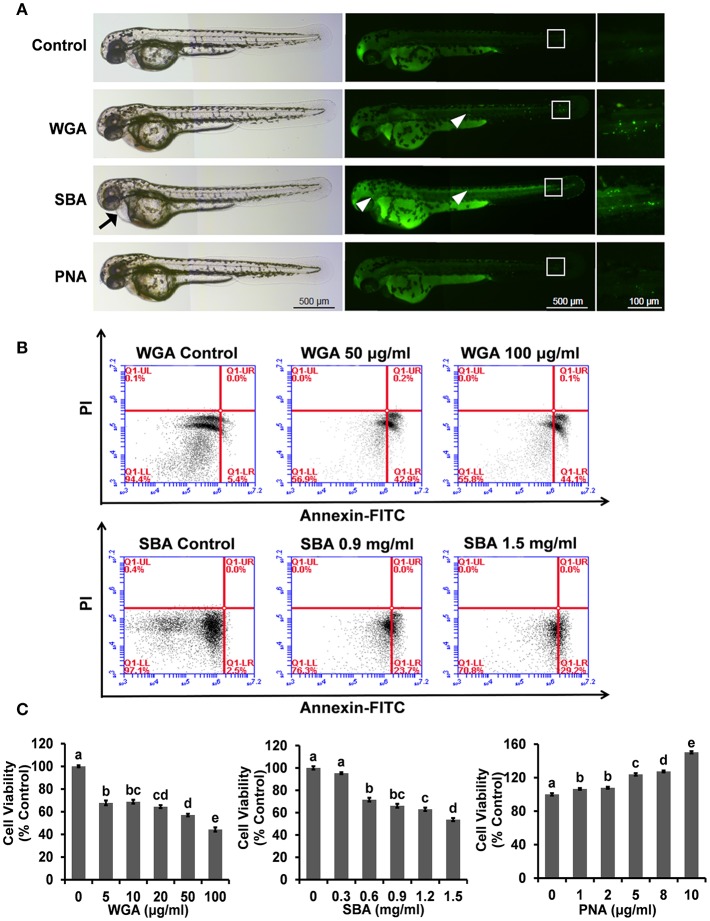
The influence of lectins on the morphogenesis of zebrafish embryos and viability and proliferation in ZFL cells. **(A)** Zebrafish embryos were treated with indicated lectins at 4 hpf. At 48 hpf, larvae were stained with AO to visualize apoptotic cells which identified as green punctate dots. Arrow indicated the pericardial edema. The boxed region indicated the tail of embryos and the magnification views were shown on the right panel. The arrowhead indicated apoptotic cells in the head and trunk region. Scale bar, 100 and 500 μm. **(B)** Cells were treated with WGA or SBA for 9 h and stained with Annexin V-FITC/PI for apoptosis analyses using flow cytometry. **(C)** Cells were treated with series concentrations of WGA or SBA for 9 h. Serum starved cells were treated with indicated concentrations of PNA for 48 h. Cell viability was evaluated by the MTT assay. Results were represented as means with standard errors (*n* = 6) and analyzed using One-Way ANOVA. Values with different letters in the same column (a–e) were significantly different (*p* < 0.05) from each other.

We first used the zebrafish embryos as an *in vivo* model to examine the whole-body effects of lectins. Compared with control group, both WGA- and SBA (500 μg/ml) treated embryos induced apoptosis in body trunk and tail parts (Figure 1A, middle and right panels). SBA-treated embryos also showed pericardial edema (Figure 1A, left panel). However, PNA-treated embryos showed no apparent effects compared to control (Figure 1A).

### The Influence of Lectins on Cell Viability and Proliferation

We further use the ZFL cell line to examine the molecular effects of lectins and their underlying mechanisms. Morphologically, cells were rounded up and detached from the culture surface after WGA treatment at 50 and 100 μg/ml for 9 h (Supplementary Figure 1A). The cell apoptotic effect of SBA was also evident at a higher dose (0.9 and 1.5 mg/ml) (Supplementary Figure 1A). The cell apoptosis was further quantitated by Annexin V-FITC and PI staining. After cells were treated with WGA at 100 μg/ml, the apoptotic ratio was raised from 5.5 ± 0.8 to 45.4 ± 0.8%. Similarly, SBA treatment at 1.5 mg/ml increased the apoptotic ratio to 29.2 ± 0.9% (Figure 1B). In contrast to the effects of WGA and SBA, PNA did not showed any effects on cell apoptosis. Rather, cell proliferation was increased after PNA treatment for 48 h. Quantitated by a MTT cell proliferation assay, cell proliferation was increased for 52.4 ± 1.4% after cells were treated with PNA at 10 μg/ml for 48 h (Figure 1C).

### The Influence of Lectins on Biomarkers of Cell Apoptosis and Proliferation

**Figure 2:**
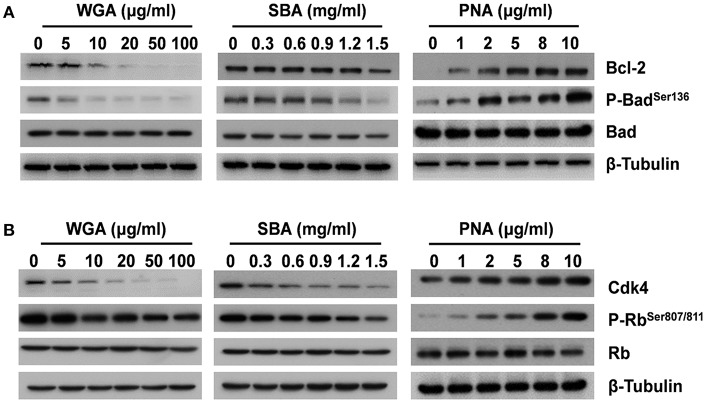
The influence of lectins on biomarkers of cell apoptosis and proliferation. Cells were treated with series concentrations of WGA or SBA for 9 h. Serum starved cells were treated with indicated concentrations of PNA for 48 h. **(A)** The levels of total and phosphorylated forms of Bad and Bcl-2 were examined by western blot. **(B)** The levels of total and phosphorylated forms of Rb, and Cdk4 were examined by western blot.

For the key molecules involved in initiating apoptosis, both the levels of anti-apoptotic B-cell lymphoma-2 (Bcl-2) and pro-apoptotic Bcl-2-associated death promoter (Bad) phosphorylation were reduced after WGA or SBA treatment (Figure 2A). In contrast, PNA increased the levels of both Bcl-2 and phospho-Bad in a dose-dependent manner (Figure 2A; Supplementary Figure 2). Furthermore, as a critical regulator for cell proliferation, the protein level of cyclin-dependent kinase 4 (Cdk4) was significantly decreased by WGA and SBA in a dose dependent manner. Accordingly, its downstream effector, the retinoblastoma (Rb) protein phosphorylation level was also reduced (Figure 2B). In contrast, PNA treatment increased the levels of both Cdk4 and phospho-Rb in a dose dependent manner (Figure 2B; Supplementary Figure 2).

### The Influence of Lectins on Intracellular Signaling

**Figure 3:**
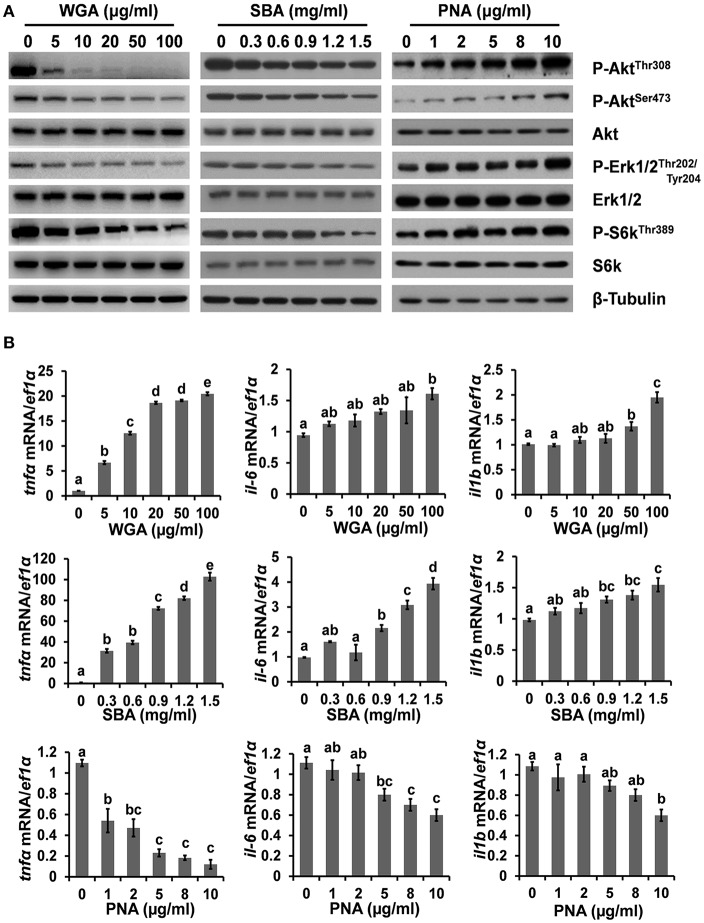
The influence of lectins on intracellular signaling. **(A)** Cells were treated with series concentrations of WGA, SBA, or PNA for 4 h and stimulated with complete medium for 15 min. The levels and activities of key signaling molecules involved in survival and stress pathways were examined by western blot. **(B)** The mRNA expressions of *il-1b, il-6*, and *tnf*α were analyzed by quantitative real-time PCR after lectins treatments. Results were represented as means with standard errors (*n* = 6) and analyzed using One-Way ANOVA. Values with different letters in the same column (a–e) were significantly different (*p* < 0.05) from each other.

To further investigate the mechanism underlying lectin-induced apoptosis or proliferation, the activities of multiple key intracellular signaling molecules were examined. WGA treatment reduced the phosphorylation levels of protein kinase B (Akt), p70 S6 kinase (S6k), and extracellular signal-regulated protein kinases 1 and 2 (Erk1/2), suggesting its inhibitory effects on these cell survival pathways (Figure 3A; Supplementary Figure 3). SBA treatment showed similar results but with higher concentrations than that of WGA. In contrast, the phosphorylation levels of Akt, S6k, and Erk1/2 were increased by PNA in a dose-dependent manner (Figure 3A). Furthermore, the mRNA expression levels of pro-inflammatory cytokines, such as interleukin-1β (*il-1b*), interleukin 6 (*il-6*), and tumor necrosis factor-α (*tnf*α), which are crucial for hepatic responses to damage and apoptosis, were all upregulated by WGA and SBA, but downregulated by PNA in dose-dependent manners (Figure 3B).

### The Influence of Lectins on Cellular Metabolism

**Figure 4:**
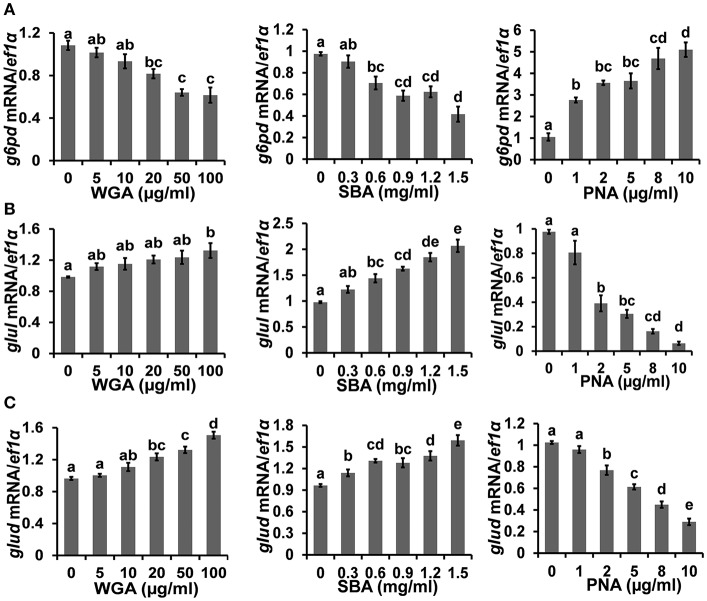
The influence of lectins on cellular metabolism. The mRNA expressions of **(A)** *g6pd*, **(B)** *glul*, and **(C)** *glud*, were analyzed by quantitative real-time PCR after lectins treatments. Results were represented as means with standard errors (*n* = 6) and analyzed using One-Way ANOVA. Values with different letters in the same column (a–e) were significantly different (*p* < 0.05) from each other.

The cellular metabolism was examined by measuring the transcriptional levels of multiple key metabolic enzymes. Firstly, the expression level of glucose-6-phosphate dehydrogenase (*g6pd*), which is critical for carbon metabolism and implicated in cell survival, was downregulated to 57.4 ± 8.8% by 100 μg/ml WGA and 42.7 ± 6.6% by 1.5 mg/ml SBA, respectively. On the contrary, the expression of *g6pd* was increased to 487.8 ± 61.7% by 10 μg/ml PNA (Figure 4A). On the other hand, the expression levels of glutamine synthetase (*glul*) and glutamate dehydrogenase (*glud*), which are key enzymes of nitrogen metabolism, were increased to 134.8 ± 10.9 and 156.4 ± 7.5%, respectively, by 100 μg/ml WGA. SBA also increased the expression levels of *glul* and *glud* to 211.7 ± 14.3 and 165.4 ± 10.4%, respectively, at 1.5 mg/ml. On the contrary, PNA treatment (10 μg/ml) reduced the expressions of *glul* and *glud* to 6.5 ± 1.3 and 28.3 ± 3.3%, respectively (Figures 4B,C).

## Discussion

There have been extensive studies on the pathological effects of plant lectins in animals (36–39), but much fewer studies were conducted to examine the cellular mechanism of lectin-mediated toxicology. In the present study, both WGA and SBA induced systemic apoptosis in zebrafish. Similar results were further confirmed in ZFL line. The apoptotic markers were subsequently examined. Specifically, Bcl-2 protein avoids the collapse of the mitochondrial transmembrane potential and inhibits the release of cytochrome c, thus prevents the activation of downstream caspase cascade that occurs during apoptosis (40, 41), while un-phosphorylated Bad binds to and inactivates Bcl-2, thus initiates apoptosis. Our results showed that both WGA and SBA reduced Bcl-2 and phospho-Bad levels, thus promoted cell apoptosis. Regardless of much neglects in normal tissues and cells, much efforts have been made to examine the apoptotic inducing effects of lectins in cancerous cells and consider it as potential anti-cancer agent (20, 21). WGA exposure induced chromatin condensation, nuclear fragmentation in human pancreatic carcinoma cells (42). SBA was also reported to cause apoptosis, autophagy, and DNA damage in Hela cells (24). Further understanding the mechanism of lectin-mediated apoptosis should be valuable for both development of anti-cancer therapies and management of food safeties.

Both WGA and SBA also elicited anti-proliferative properties in the present study, as demonstrated by both MTT cell proliferation assay and cell cycle biomarkers. Cdk4 is known to phosphorylate and inhibit members of the retinoblastoma protein and critical for G1 to S phase progression (43, 44). WGA and SBA reduced the levels of Cdk4 and phospho-Rb in a dose-dependent manner. Similarly, WGA was also found to cause cell cycle arrest in mouse fibroblast cells (45). Another study reported decreased mRNA expression of cell cycle-related gene including Cdk4 in intestinal epithelial cell line from piglets (46). The combinatory pro-apoptotic and anti-proliferative effects of WGA and SBA should thus delineate their toxicity for cells and organs, with WGA being more toxic at lower doses than SBA. In contrast to the effects of WGA and SBA, PNA showed no toxic effects on zebrafish development and cells even at high doses (up to 1 mg/ml). Rather, PNA was found to stimulate cell proliferation. These effects were further corroborated by the increased levels of anti-apoptotic and cell cycle progression markers. There were reports showed that PNA had stimulatory (47), no (48), and inhibitory (49) effects on the proliferation of different cancerous cell lines, but the mechanism remains unknown.

Previous studies, including those conducted in cancerous cell lines, were mainly focused on the pathological and apoptotic inducing effects of lectins (6, 20, 23). The upstream cell signaling pathways influenced by lectins were far less characterized. In the present study, the effects of lectins on major cell survival and proliferation pathways were examined. Phosphatidylinositide 3 kinases (PI3Ks) and their downstream mediators AKT and mechanistic target of rapamycin (TOR) constitute the core signaling cascade regulating cell proliferation, survival, and metabolism (50–52). Phosphorylated AKT induces proliferation and inhibits apoptosis by phosphorylating of several target proteins involved in apoptosis, such as BAD (53). Upon activation, mTOR regulates many cellular functions, such as cell growth, protein synthesis, and autophagy (54). ERK1/2 are members of the mitogen-activated protein kinase super (MAPK) family that mediate cell proliferation and apoptosis through phosphorylating of downstream apoptosis-related molecules (55). In the present study, the activities of Akt, Tor, and Erk1/2 signaling were inhibited by WGA and SBA, while stimulated by PNA in contrast. This was consistent with previous report demonstrated that the mitogenic effect of PNA in colonic epithelial cells was mediated by stimulation of MAPK/ERK signaling pathway (56). A previous study also showed that high concentrations of PNA (up to 100 μg/ml) selectively induced reactive oxygen species (ROS) production and apoptosis in multiple cancer cell lines, but not in normal cells (57). Furthermore, we found that WGA and SBA induced the expression of pro-inflammatory cytokines (*tnf*α, *il-6*, and *il-1*β), which were known to dephosphorylate Bad and stimulate apoptosis (58). Promoted secretion of pro-inflammatory cytokines by certain plant lectins might contribute to food-associated chronic inflammation and inflammatory diseases (59, 60).

The cell survival and proliferation are intricately integrated with cellular metabolic status (61). The pentose phosphate pathway (PPP) and its rate-limiting enzyme, *g6pd*, are responsible for NADPH generation, which protects cells from oxidative damage and acts as an essential cofactor for the reductive biosynthesis of nucleotides, amino acids, and fatty acids (62, 63). As such, the upregulation of PPP activity is a hallmark of rapidly proliferating cells (64). In the present study, the expression level of *g6pd* was down-regulated by WGA and SBA, but up-regulated by PNA, thus echoing the corresponding cellular status under these treatments. On the other hand, WGA and SBA also increased the expression levels of *glud* and *glul*, which could be up-regulated upon PI3K-Akt signaling inhibition (65) and stimulated the cellular use of glutamate or glutamine as substrates for ATP production (66, 67). Indirectly feeding the Krebs cycle and the subsequent respiratory chain by glutamine metabolism in turn stimulated the production of reactive oxygen species (ROS), which is apoptotic inductive (68–70).

As shown in Supplementary Figure 4, the primary sequence, three-dimensional structure, and carbohydrate recognition specificities of these lectins were very different from each other. It is very difficult to correlate their structures with respected functions of these lectins only based on the limited knowledge available so far. Further studies were much warranted to elucidate the underlying mechanism of their cellular effects.

In conclusion, the present study demonstrated the differential impacts of lectins (WGA, SBA, and PNA) from three commonly used plant protein sources (wheat, soybean, and peanut meals) toward embryonic development and liver cells of zebrafish. Supported by cell signaling and metabolic analyses, we demonstrated that both WGA and SBA caused apoptosis while PNA stimulated cell proliferation in contrast. The present study cautions the general consideration of plant lectins toxic without discrimination. Further characterization of the distinct functions of individual lectins should be valuable for both nutrition and other potential applications.

## Contribution to the Field Statement

Lectins are major anti-nutritional factors in plant proteins and toxic to human and animals. However, the cellular effects and causative mechanism are far from fully understood for particular lectins. In the present study, we chose lectins from three common sources, wheat (WGA), soybean (SBA), and peanut (PNA). Their effects were examined in zebrafish embryo and liver cells. Both WGA and SBA caused apoptosis, while PNA stimulated cell proliferation. A full examination of cell signaling and metabolic biomarkers was conducted to reveal the underlying mechanism. This study represents a rare approach that examines the effects of lectins in fish and provides a comprehensive mechanistic characterization. The results should be valuable for better utilization of plant proteins in aquatic animals.

## Author Contributions

GH and KM designed the research. GH, KW, and CL conducted the research, analyzed the data, and wrote the paper. YH, XW, and HZ provided technical assistance and contributed to the preparation of the figures. All authors read and approved the final manuscript.

### Conflict of Interest Statement

The authors declare that the research was conducted in the absence of any commercial or financial relationships that could be construed as a potential conflict of interest.
