# Supplementary material for: Differential Apoptotic and Mitogenic Effects of Lectins in Zebrafish
Source: Front Endocrinol (Lausanne). 2019 Jun 5;10:356. doi: 10.3389/fendo.2019.00356 (PMC6560201; doi:10.3389/fendo.2019.00356)
Supplement: Supplementary file 1 [file Data_Sheet_1.pdf]

Supplementary Figure 1

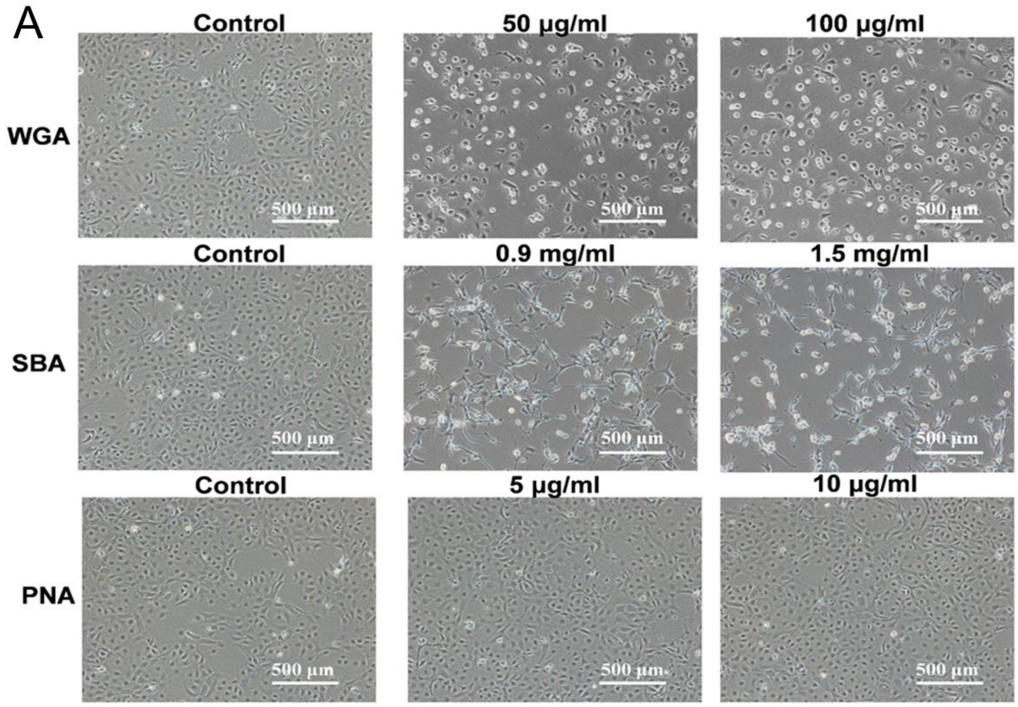

Supplementary Figure 1. (A) Representative morphology of ZFL cells was detected after lectins treatments. Scale bar, 500 µm.

Supplementary Figure 2

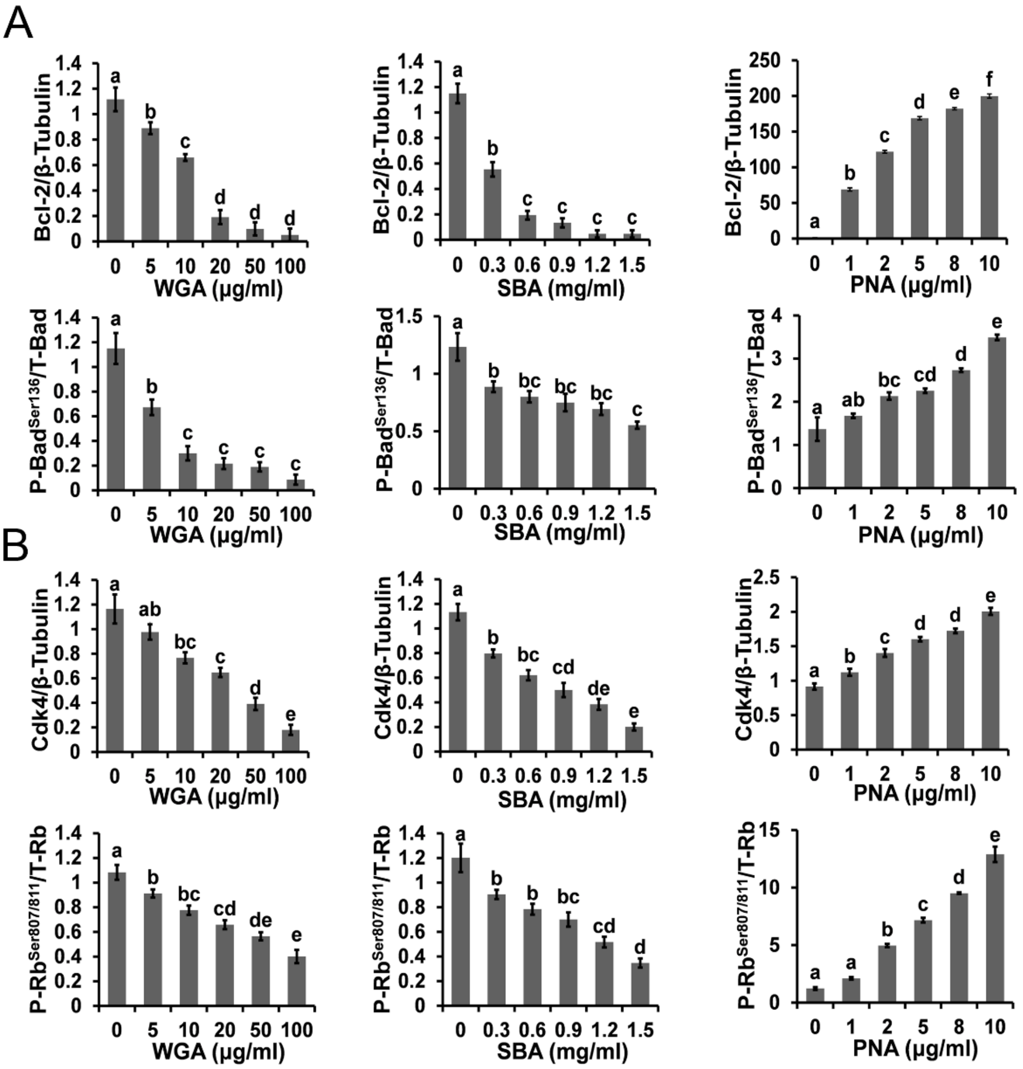

Supplementary Figure 2. (A) The quantitative data of Figure 2A. **(B)** The quantitative data of Figure 2B. Results were represented as means with standard errors (n=3) and analyzed using One-Way ANOVA. Values with different letters in the same column (a-f) were significantly different ($p < 0.05$) from each other.

Supplementary Figure 3

A

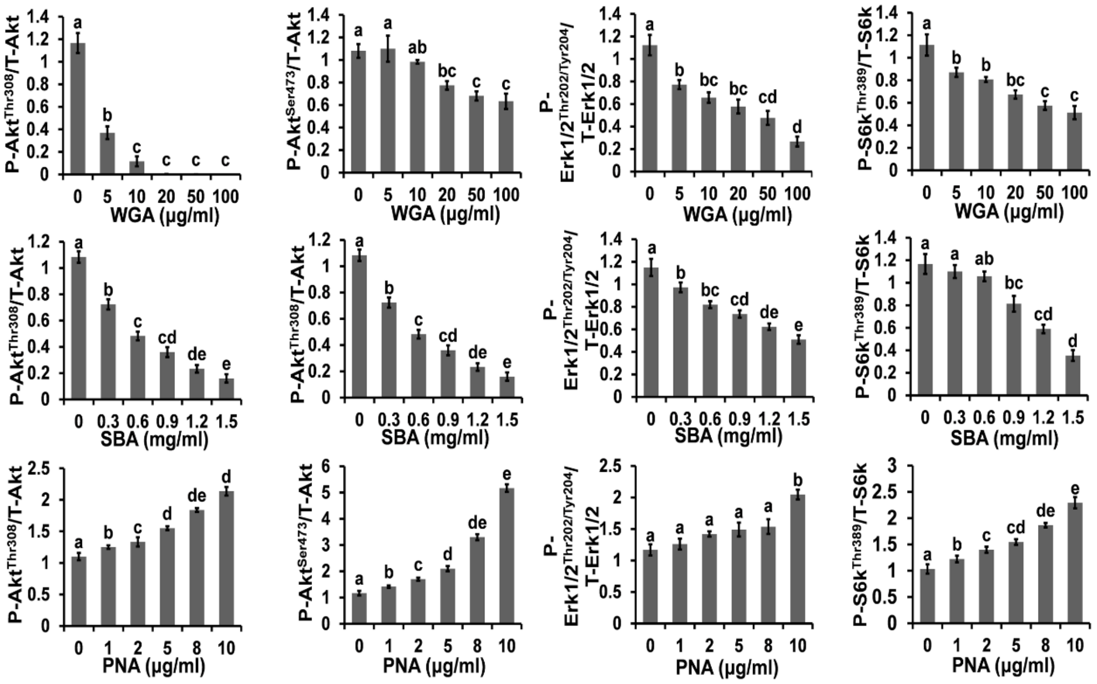

Supplementary Figure 3. (A) The quantitative data of Figure 3A. Results were represented as means with standard errors (n=3) and analyzed using One-Way ANOVA. Values with different letters in the same column (a-d) were significantly different ($p < 0.05$) from each other.

Supplementary Figure 4

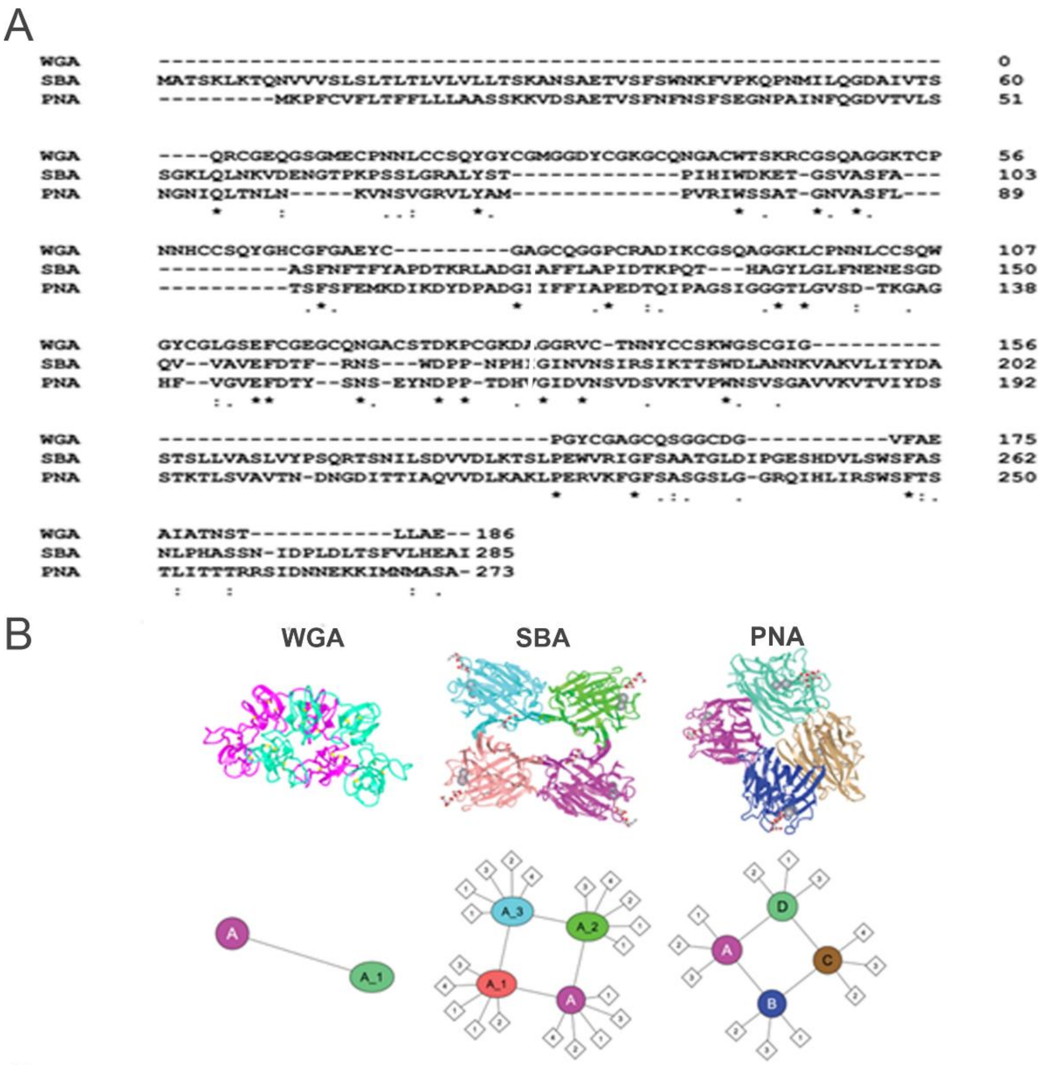

C

The carbohydrate specificity of WGA, SBA and PNA.

Lectin	Monosaccharides	Glycans
WGA	GlcNAc (low affinity)	Dimers, Trimers, oligosaccharides and glycoproteins containing GlcNAc, NeuNAc, (β1-4GlcNAc)1-2NeuNAc GlcNAcβ1-4GlcNAcβ1-4GlcNAcβ1-4GlcNAcβ1-4GlcNAc
SBA	GalNAc/Gal	Oligosaccharides with terminal α- or β-linked NAcGal/Gal residues
PNA	Gal	Galβ1-3GalNAc, Lactose Galβ1-3GalNAc-Ser/Thr

Supplementary Figure 4. (A) Sequence alignment of WGA (partial, AAA34257.1), SBA (NP_001341753), and PNA (XP_025677571) were performed using Clustal Omega. **(B)** 3D crystal structures of WGA (PDB ID: 9WGA), SBA (PDB ID: 1SBE), and PNA (PDB ID: 2PEL). The interacted chemicals are indicated. The lower panels show protein-protein and protein-chemicals interactions. The chemicals interacted with

SBA are: 1. N-Acetyl-D-Glucosamine; 2. Beta-D-Galactose; 3. Manganese Ion; 4. Beta-Lactose. The chemicals interacted with PNA are: 1. Alpha-Lactose; 2. Calcium Ion; 3. Manganese Ion; 4. Beta-Lactose. **(C)** The carbohydrate specificity of WGA, SBA and PNA. Based on: Maldonado, et al. *Adv Biosci Biotechnol* (2013) 4:67-74.

Supplementary Table 1. Primer sequences used for real-time quantitative PCR.

Gene	Forward Primer (5'-3')	Reverse Primer (5'-3')	product size (bp)
glul	TGTGAAGACTTCGGTGTGGT	TGCGGATGTGATAGTTGTGC	175
glud	CCATCCCTATTGTACCCACTG	TTGACGTAAGCCGCTGTTC	172
g6pd	GCCTCCCTTCAGCACATAGA	ATGGGGATGCCCTCGTATT	170
il-1b	CTGAAATGATGGCATGCGGG	TGCAAGCGGATCTGAACAGT	111
il-6	TCAACTTCTCCAGCGTGATG	TCTTTCCCTCTTTTCCTCCTG	73
tnfα	GCTGGATCTTCAAAGTCGGGTGTA	TGTGAGTCTCAGCACACTTCCATC	81
ef1α	TGTCCTCAAGCCTGGTATGG	TGGGTCGTTCTTGCTGTCTC	190

Abbreviations and GenBank Accession No.: *glul*, glutamine synthetase, NM_181559; *glud*, glutamate dehydrogenase, NM_199545; *g6pd*, glucose-6-phosphate dehydrogenase, XM_692728.6; *il-6*, interleukin 6, NM_001114318; *il-1b*, interleukin-1β, AY340959; *tnfα*, tumor necrosis factor-α, NM_212859; *ef1α*, elongation factor-1α, XM_005173785.
